# Supplementary material for: Evolutionary histories of breast cancer and related clones
Source: Nature. 2023 Jul 26;620(7974):607–14. doi: 10.1038/s41586-023-06333-9 (PMC10432280; doi:10.1038/s41586-023-06333-9)
Supplement: Supplementary file 1 — This file contains Supplementary Notes 1 and 2 and includes Figs. 1–4. [file 41586_2023_6333_MOESM1_ESM.pdf]

nature portfolio

https://doi.org/10.1038/s41586-023-06333-9

Supplementary information

Evolutionary histories of breast cancer and related clones

In the format provided by the authors and unedited

Supplementary Note 1. Estimation of sensitivity of WGS mutation calling

For single cell-derived organoids (n=71), the sensitivity of WGS mutation calling was estimated by calculating the fraction of unique heterozygous germline polymorphisms in each sample detected via paired analysis using another participant's germline sample to mimic a matched control **(Supplementary Fig. 1)**. Regarding FFPE and fresh-frozen LCM samples, sequencing reads of a LCM sample was merged with those of another participant' s germline sample at different mixing rates to mimic the tumour samples containing 10–100% of tumour cells with the target coverage; the sensitivity was estimated using polymorphisms on the chromosomal segments without a copy number change in five and three samples for FFPE and fresh-frozen LCM samples, respectively **(Supplementary Figs. 2,3)**. The estimated sensitivity is summarised in **Supplementary Table 13**.

Supplementary Figure 1: Estimated sensitivity of WGS mutation calling in single cell-derived organoids

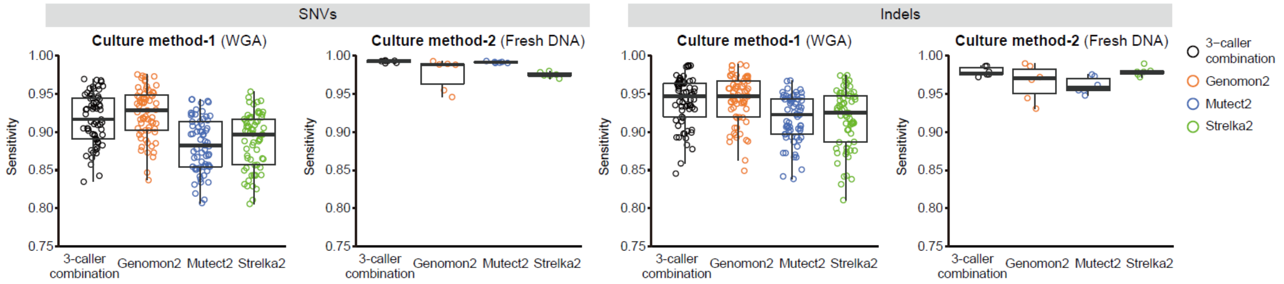

Supplementary Figure 1 legend:

Estimated sensitivity of WGS mutation calling in whole-genome amplified (WGA) organoid samples established via culture method-1 (*n*=65) and fresh DNA samples extracted from organoids established via culture method-2 (*n*=6) in **Extended Data Fig. 1a**. Sensitivity was estimated for four different calling algorithms; '3-caller combination' is the method used in our entire analysis, wherein the mutations called via ≥2 of 3 callers (Genomon2, Mutect2, and Strelka2) were considered 'high confidence' mutation calls (**Online Methods**). Box plots show the median values and hinges corresponding to the first and third quartiles, with whiskers that extend to the furthest value within a 1.5× interquartile range.

Supplementary Figure. 2: Estimated sensitivity of WGS mutation calling in FFPE LCM samples

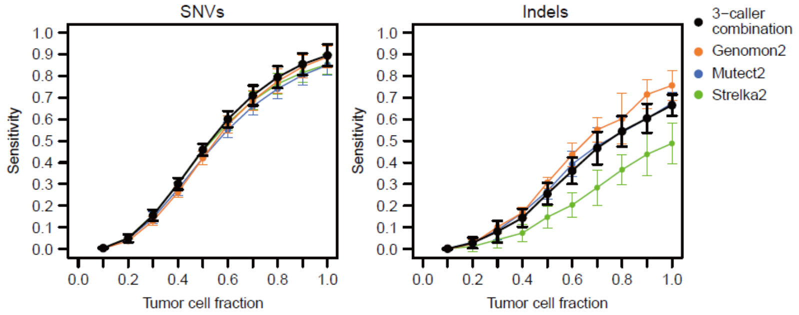

Supplementary Figure. 2 legend:

Average of estimated sensitivity of WGS mutation calling for each mixing rate in FFPE LCM samples (*n*=5), with error bars indicating ± standard deviation. Sensitivity was estimated for four different calling algorithms; '3-caller

combination' is the method used in our entire analysis, wherein the mutations called via ≥2 of 3 callers (Genomon2, Mutect2, and Strelka2) were considered 'high confidence' mutation calls.

Supplementary Figure 3: Estimated sensitivity of WGS mutation calling in fresh-frozen LCM samples

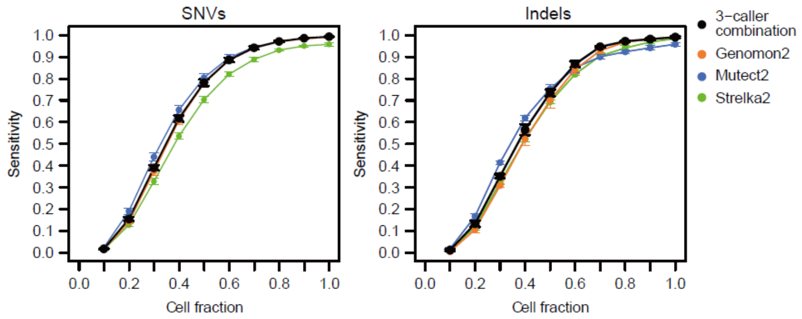

Supplementary Figure 3 legend:

Average of estimated sensitivity of WGS mutation calling for each mixing rate in fresh-frozen LCM samples (*n*=3), with error bars indicating ± standard deviation. Sensitivity was estimated for four different calling algorithms; '3-caller combination' is the method used in our entire analysis, wherein the mutations called via ≥2 of 3 callers (Genomon2, Mutect2, and Strelka2) were considered 'high confidence' mutation calls.

Supplementary Note 2. Validation of the phylogenetic analysis

Phylogenetic trees were reconstructed by combining somatic mutation data and copy number information across all LCM samples using MEGA and 'treemut.' To validate this approach, we reconstructed trees for the representative two cases (KU779 **(Fig. 2a)** and KU539 **(Extended Data Fig. 5a)**) using another software, PyClone-VI (v0.1.0, URL: https://github.com/Roth-Lab/pyclone-vi), which estimates clusters of mutations shared by clones or subclones, or 'branch' in the tree, and the samples that share each cluster on the basis of mutant cell fraction, the number of mutant and wild-type reads, and total and minor copy number at the mutation locus for all mutations in all samples. These clusters were then visually ordered so that the samples in a parent branch comprise those in their child branches across all branchpoints. Here, mutation clusters were excluded if the sum of the mutant fraction of parent and child branches did not exceed 1.0 according to the Pigeonhole principle. When clusters showed inconsistency, clusters of fewer mutations were excluded so that the remaining clusters remained consistent.

Because PyClone has a limitation for the number of samples to be analysed accurately, we first focused on the top three branchpoints of the tree reconstructed using MEGA/treemut. We randomly selected a sample if a branch comprised a set of multiple samples, which were analysed separately. As for multiple samples from a single tumour focus, one sample was randomly selected among them.

Topology of the trees was largely concordant (22/25, 88%) between MEGA/treemut- and PyClone-VI-reconstructed trees, with a few exceptions at peripheral branches **(Supplementary Fig. 4)**. The assignment of driver mutations to branches was 100% concordant between them.

Supplementary Figure. 4: Trees reconstructed using MEGA/treemut and PyClone-VI

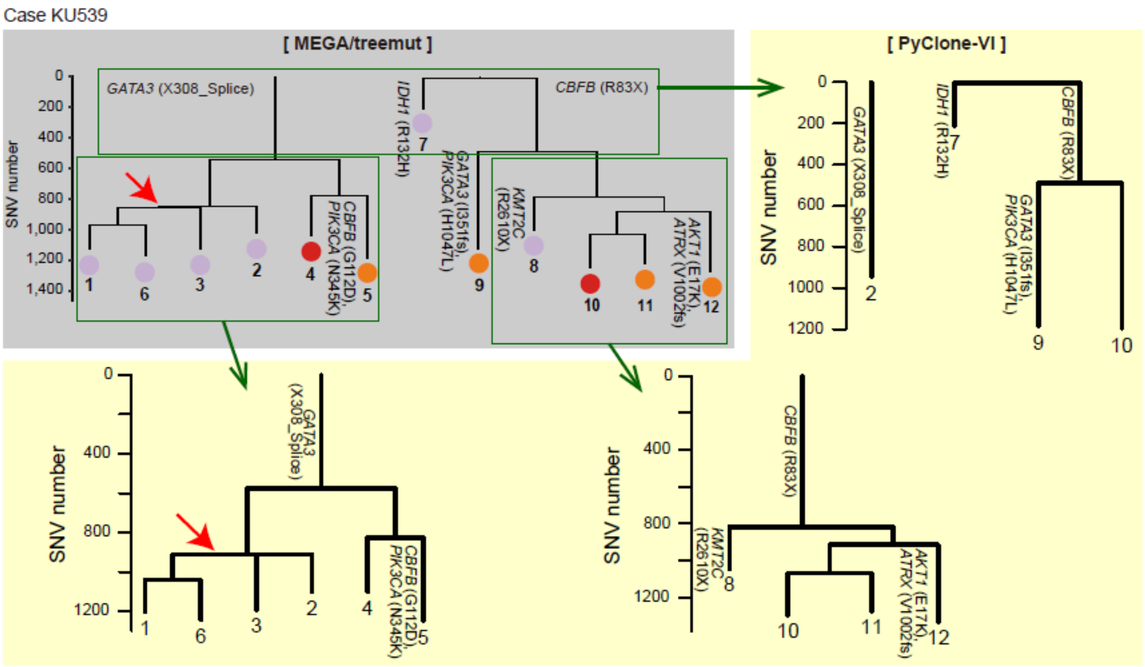

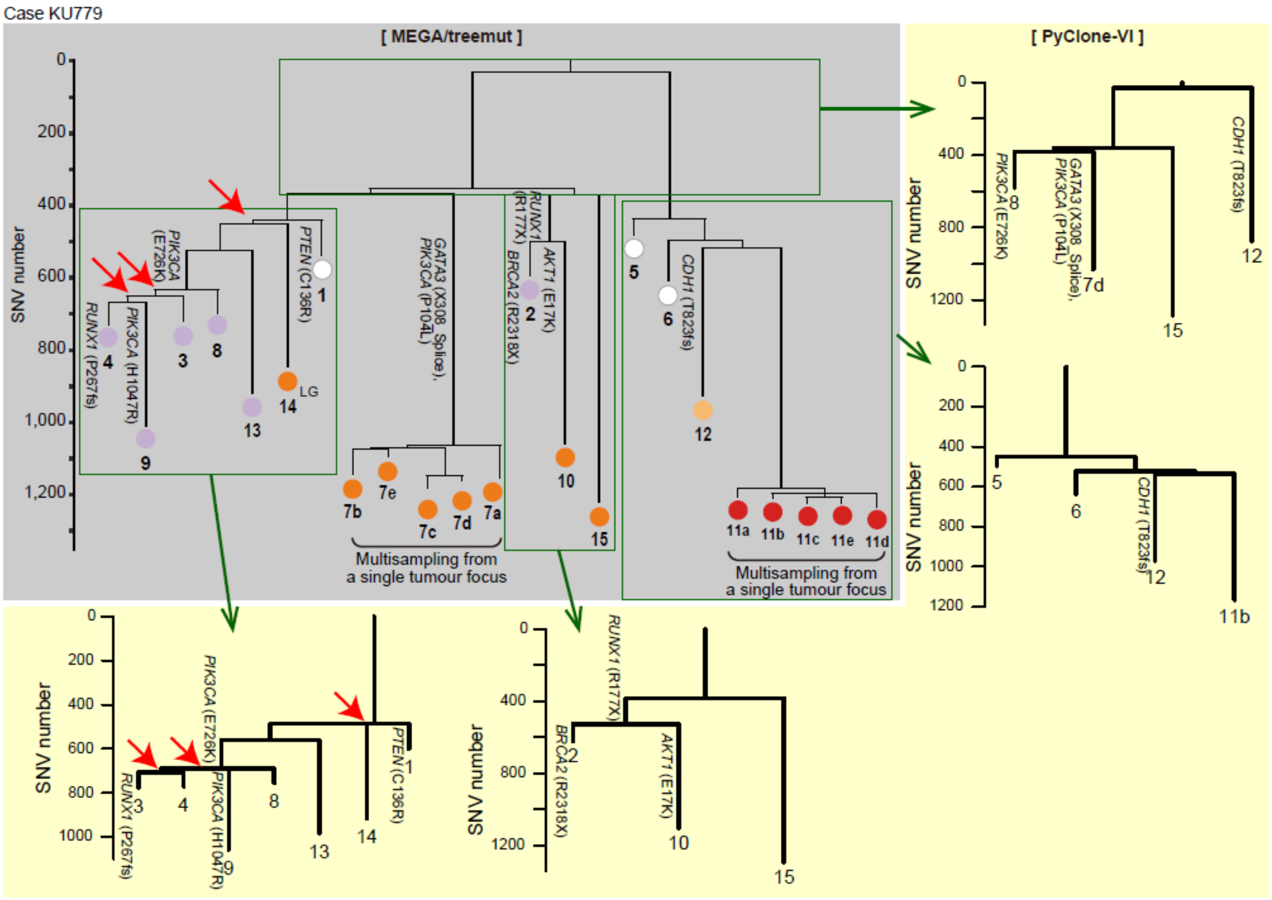

Supplementary Figure. 4 legend:

Trees reconstructed using PyClone-VI are shown as well as those reconstructed using MEGA/treemut. The nodes which were not concordant between the trees are indicated by red arrows.
