# Supplementary material for: Evolutionary histories of breast cancer and related clones
Source: Nature. 2023 Jul 26;620(7974):607–14. doi: 10.1038/s41586-023-06333-9 (PMC10432280; doi:10.1038/s41586-023-06333-9)
Supplement: Supplementary file 2 — Reporting Summary [file 41586_2023_6333_MOESM2_ESM.pdf]

nature portfolio

Corresponding author(s): Seishi Ogawa

Last updated by author(s): Jun 5, 2023

Reporting Summary

Nature Portfolio wishes to improve the reproducibility of the work that we publish. This form provides structure for consistency and transparency in reporting. For further information on Nature Portfolio policies, see our Editorial Policies and the Editorial Policy Checklist.

Statistics

For all statistical analyses, confirm that the following items are present in the figure legend, table legend, main text, or Methods section.

n/a	Confirmed	
☐	☒	The exact sample size (*n*) for each experimental group/condition, given as a discrete number and unit of measurement
☐	☒	A statement on whether measurements were taken from distinct samples or whether the same sample was measured repeatedly
☐	☒	The statistical test(s) used AND whether they are one- or two-sided<br>*Only common tests should be described solely by name; describe more complex techniques in the Methods section.*
☐	☒	A description of all covariates tested
☐	☒	A description of any assumptions or corrections, such as tests of normality and adjustment for multiple comparisons
☐	☒	A full description of the statistical parameters including central tendency (e.g. means) or other basic estimates (e.g. regression coefficient) AND variation (e.g. standard deviation) or associated estimates of uncertainty (e.g. confidence intervals)
☐	☒	For null hypothesis testing, the test statistic (e.g. *F*, *t*, *r*) with confidence intervals, effect sizes, degrees of freedom and *P* value noted<br>*Give P values as exact values whenever suitable.*
☒	☐	For Bayesian analysis, information on the choice of priors and Markov chain Monte Carlo settings
☒	☐	For hierarchical and complex designs, identification of the appropriate level for tests and full reporting of outcomes
☐	☒	Estimates of effect sizes (e.g. Cohen's *d*, Pearson's *r*), indicating how they were calculated

Our web collection on statistics for biologists contains articles on many of the points above.

Software and code

Policy information about availability of computer code

Data collection	No software was used for data collection.
Data analysis	Detailed descriptions of the software and analysis have been provided in Online Methods. Sequencing data was processed using Genomon2. External bam files were converted to fastq format using biobambam. Mutation calling was performed using Genomon2, Mutect2 (GATK4), and Strelka2. Copy number analysis using sequencing data was performed using Control-FREEC and in-house pipeline CNACS. Number of clonal mutations in single cell-derived organoids was estimated using mclust. Phylogenetic analysis was performed using MEGA, treemut, and PyClone-VI. Mutational signature was evaluated using MutationalPatterns, SigProfiler Bioinformatic Tools MatrixGenerator and Extractor, HDP, and deconstructSigs. Significantly mutated genes were identified using dndscv. Statistical analyses were performed using R (3.6.3). Survival analysis was performed using the R package survival.<br><br>CNACS is deposited in GitHub (https://github.com/OgawaLabTumPath/CNACS).<br><br>List of programs and softwares:<br>Genomon2 pipeline: version 2.6.2 (https://genomon.readthedocs.io/ja/latest/)<br>- Burrows-Wheeler Aligner: version 0.7.8 (https://sourceforge.net/projects/bio-bwa/)<br>- biobambam: version 0.0.191 (https://www.sanger.ac.uk/science/tools/biobambam)<br>- GenomonMutationFilter: version 0.2.1 (https://github.com/Genomon-Project/GenomonMutationFilter)<br>Xenome: version 1.0.0 (https://github.com/data61/gossamer)<br>Samtools: version 1.10 (https://github.com/samtools/samtools)<br>GATK4: version 4.1.2 (https://github.com/broadinstitute/gatk/releases)<br>Strelka2: version 2.9.3 (https://github.com/Illumina/strelka)

ANNOVAR: 2020-06-07 (https://annovar.openbioinformatics.org/en/latest/)
Integrative Genomics Viewer (IGV): version 2.3.8 (http://software.broadinstitute.org/software/igv/)
Control-FREEC: version 11.0 (https://github.com/BoevaLab/FREEC/releases)
mclust: version 5.4.7 (https://cran.r-project.org/web/packages/mclust/index.html)
MEGA: version 11.0.11 (https://www.megasoftware.net/)
treemut: version 1.1 (https://github.com/NickWilliamsSanger/treemut)
PyClone-VI: version 0.1.0 (https://github.com/Roth-Lab/pyclone-vi)
MutationalPatterns: version 3.4.0 (https://bioconductor.org/packages/release/bioc/html/MutationalPatterns.html)
SigProfiler Bioinformatic Tools MatrixGenerator: version 1.1.27 (https://github.com/AlexandrovLab/SigProfilerMatrixGenerator)
SigProfiler Bioinformatic Tools Extractor: version 1.1.1 (https://github.com/AlexandrovLab/SigProfilerExtractor)
HDP: version 0.1.5. (https://github.com/nicolaroberts/hdp)
deconstructSigs: version 1.8.0 (https://github.com/raerose01/deconstructSigs)
dndscv: version 0.0.1.0 (https://github.com/im3sanger/dndscv)
R: version 3.6.3 (https://cran.r-project.org/)
survival: version 3.2.11 (https://cran.r-project.org/web/packages/survival/index.html)

The R codes for phylogenetic analysis and estimation of the timing of the MRCA emergence and der(1;16) acquisition are available in Supplementary Notes 3 and 4, respectively.
The R codes for estimation of mutation rate in normal cells are available at https://doi.org/10.5281/zenodo.8002434.

For manuscripts utilizing custom algorithms or software that are central to the research but not yet described in published literature, software must be made available to editors and reviewers. We strongly encourage code deposition in a community repository (e.g. GitHub). See the Nature Portfolio guidelines for submitting code & software for further information.

Data

Policy information about availability of data

All manuscripts must include a data availability statement. This statement should provide the following information, where applicable:

- Accession codes, unique identifiers, or web links for publicly available datasets
- A description of any restrictions on data availability
- For clinical datasets or third party data, please ensure that the statement adheres to our policy

All WGS data have been deposited in the European Genome-phenome Archive (http://www.ebi.ac.uk/ega/) under accession number EGAS00001006282.
Data for estimation of mutation rate and phylogenetic analysis are available at https://doi.org/10.5281/zenodo.8002434.
Data for the Figures and Extended Data Figures are available as Source Data.

WES bam files, RNAseq data in the TPM format, and clinicopathological information of TCGA datasets were downloaded from TCGA data portal (https://portal.gdc.cancer.gov/), whereas the information about PAM50 mRNA subtypes was extracted from the study by Ciriello et al. (DOI: 10.1016/j.cell.2015.09.033); if data were lacking, information was extracted from TCGA Network (DOI: 10.1038/nature11412).
The publicly available GRCh37 (hg19, https://www.ncbi.nlm.nih.gov/assembly/GCF_000001405.13/) was used as human reference genome in this study.
We referred to the 1000 Genomes Project dataset (1000g2015aug, downloaded through ANNOVAR (DOI:10.1093/nar/gkq603)), the gnomAD database (gnomad_genome, downloaded through ANNOVAR), the GenomicSuperDups database (downloaded through ANNOVAR), repetitive sequences reported in the UCSC Genome Browser (DOI:10.1101/gr.229102), COSMIC (the Catalogue Of Somatic Mutations In Cancer) database (https://cancer.sanger.ac.uk/cosmic), and ClinVar database (https://www.ncbi.nlm.nih.gov/clinvar/), for variant annotation.
COSMIC SBS signatures (v3.1) were obtained from COSMIC (https://cancer.sanger.ac.uk/signatures/downloads/).

Human research participants

Policy information about studies involving human research participants and Sex and Gender in Research.

Reporting on sex and gender	We analysed specimens derived from female breast cancer patients and healthy women to investigate clonal evolution of normal mammary epithelial cells into breast cancer in women.
Population characteristics	We enrolled 207 female breast cancer patients who underwent surgery at the Kyoto University Hospital, aged 26 to 92, and eight healthy breastfeeding women who delivered at the Kyoto University Hospital or Adachi Hospital, aged 22 to 37. The characteristics of the participants are summarised in Supplementary Table 1.
Recruitment	To estimate the rate of mutation accumulation in normal mammary epithelial cells, we enrolled 15 sporadic female breast cancer patients who underwent total mastectomy at the Kyoto University Hospital, and eight healthy breastfeeding women with adequate breast milk supply who had delivered at the Kyoto University Hospital or Adachi Hospital. These participants were recruited at random.<br><br>For the analysis of cancer-related clonal evolution, All 156 female breast cancer patients who underwent surgery without any preoperative treatment at the Kyoto University Hospital from 2015 to 2017 and agreed to offer surgical specimens were recruited. Next, they were screened based on the pathology reports to select the cases with multiple large non-cancerous proliferative lesions near cancers. In total, five sporadic cases with available archival FFPE surgical specimens were found, and all the cases were analysed via sequencing. The details of case selection are shown in Online methods. We dared to select breast cancers accompanied by multiple proliferative lesions to explore life history of breast cancer, which resulted in the enrichment of der(1;16)(+) cancers.<br>To further evaluate the pathological feature of der(1;16)(+) breast cancers, we obtained 33 breast cancer tissue cores (28 Luminal A-like invasive cancers and 5 ER(+)HER2(-) non-invasive cancers) in the tissue microarray provided by the Kyoto

Breast Cancer Research Network (KBCRN) BORN (Breast Oncology Research Network)-BioBank, which was established using surgical specimens of randomly recruited cases. Two premenopausal and six postmenopausal der(1;16)(+) cancer cases were found by FISH analysis, and they all were evaluated in this study. The details of case selection are also shown in Online Methods.

For the analysis of non-cancer clones unrelated to cancer, we enrolled 3 premenopausal breast cancer patients who underwent total mastectomy without any preoperative treatment at the Kyoto University Hospital, to obtain enough amount of normal epithelium before atrophy due to menopause. The patients were recruited at random.

Ethics oversight: This study was reviewed and approved by the ethics committees of the Kyoto University and Adachi Hospital.

Note that full information on the approval of the study protocol must also be provided in the manuscript.

Field-specific reporting

Please select the one below that is the best fit for your research. If you are not sure, read the appropriate sections before making your selection.

☒ Life sciences ☐ Behavioural & social sciences ☐ Ecological, evolutionary & environmental sciences

For a reference copy of the document with all sections, see nature.com/documents/nr-reporting-summary-flat.pdf

Life sciences study design

All studies must disclose on these points even when the disclosure is negative.

Sample size	No statistical methods were used to determine sample size since this is an exploratory study. We enrolled as many patients as possible who provided consent for our study during the enrollment period between 1/Jan/2015 and 1/Dec/2020.
Data exclusions	For the analysis of TCGA BRCA cohort, we excluded whole-genome amplified samples to avoid artefactual mutation callings. We also excluded samples with pathogenic germline variants and samples with low quality copy number data, to evaluate the association of specific copy number events, clinicopathological information, and somatic mutation profiles in the sporadic BRCA cohort.
Replication	We did not attempt replication in this study, except for WGS of WGA organoid samples, in which two independent experiments were performed to eliminate WGA-related sequencing errors. The details of sequencing methods for WGA samples are described in Online Methods.<br><br>As for sequencing experiments, we validated the results by confirmatory targeted capture sequencing for variants (n = 702) from 22 samples randomly selected from 126,653 variants from 228 samples. We also validated variants detected in the most common recent ancestors in FFPE multi-sampled cases as many as possible (n = 10,190 out of 10,629 variants) in 27 samples from five cases (out of 84 samples from 10 cases) to ensure the accuracy of phylogenetic tree reconstruction and the subsequent estimation of timing for initial events. The details of validation sequencing are described in Online methods and Supplementary Tables 14 and 15.
Randomization	Not applicable since this is a case-series study which was therefore not planned to detect any difference in effects between the cohorts with and without intervention.
Blinding	Pathologists were blinded to the genetic alterations in each sample during histopathological evaluation.

Reporting for specific materials, systems and methods

We require information from authors about some types of materials, experimental systems and methods used in many studies. Here, indicate whether each material, system or method listed is relevant to your study. If you are not sure if a list item applies to your research, read the appropriate section before selecting a response.

Materials & experimental systems

n/a	Involved in the study	
☐	☒	Antibodies
☒	☐	Eukaryotic cell lines
☒	☐	Palaeontology and archaeology
☒	☐	Animals and other organisms
☒	☐	Clinical data
☒	☐	Dual use research of concern

Methods

n/a	Involved in the study	
☒	☐	ChIP-seq
☒	☐	Flow cytometry
☒	☐	MRI-based neuroimaging

Antibodies

Antibodies used: ER (supplier name, Roche Diagnostics; catalogue number, 790-4325; clone name, SP1)

Antibodies used

PR (supplier name, Roche Diagnostics; catalogue number, 790-2223; clone name, 1E2)
HER2 (supplier name, Roche Diagnostics; catalogue number, 790-2991; clone name, 4B5)
Ki-67 (supplier name, Agilent Technologies; catalogue number, M7240; clone name, MIB-1)
CD326 (EpCAM) MicroBeads human (supplier name, Miltenyi Biotec; catalogue number, 130-061-101)
CD45 Microbeads human (supplier name, Miltenyi Biotec; catalogue number, 130-045-801)
pan-cytokeratin antibody cocktails (AE1/AE3) (supplier name, NICHIREI; catalogue number, 412811)
CK5 (supplier name, Leica Biosystems; catalogue number, CK5-L-CE-H; clone name, XM26)
E-cadherin (supplier name, Agilent Technologies; catalogue number, M3612; clone name, NCH-38)

Validation

ER (human; IHC; validation: Manufacturer - https://pim-eservices.roche.com/eLD/web/pi/en/documents/download/2b989f55-4533-ea11-fc90-005056a71a5d)
PR (human; IHC; validation: Manufacturer - https://pim-eservices.roche.com/eLD/web/pi/en/documents/download/76ea4fea-e112-ea11-fa90-005056a772fd)
HER2 (human; IHC; validation: Manufacturer - https://pim-eservices.roche.com/eLD/web/pi/en/documents/download/fc569ff5-2236-ea11-fc90-005056a71a5d)
Ki-67 (human; IHC; validation: Manufacturer - http://webzis.cytopathos.sk/Protilatky/store/MIB1.pdf)
CD326 (EpCAM) MicroBeads human (human; MicroBeads conjugated to monoclonal antibody: validation, Manufacturer - http://www.ulab360.com/files/prod/manuals/201603/28/596760001.pdf)
CD45 Microbeads human (human; MicroBeads conjugated to monoclonal antibody; validation: Manufacturer - https://www.miltenyibiotec.com/upload/assets/IM0001290.PDF)
pan-cytokeratin antibody cocktails (AE1/AE3) (human; IHC; validation: Tohyama R, Kayamori K, Sato K, et al. Establishment of a xenograft model to explore the mechanism of bone destruction by human oral cancers and its application to analysis of role of RANKL. J Oral Pathol Med. 2016; 45(5):356-64.)
CK5 (human; IHC; validation: Manufacturer - https://shop.leicabiosystems.com/ja-jp/actions/ViewProductAttachment-OpenFile?LocaleId=en_US&DirectoryPath=SDSs&FileName=ck5-l-ce.pdf&UnitName=LBS)
E-cadherin (human; IHC; validation: Manufacturer - http://webzis.cytopathos.sk/Protilatky/store/Cadherin%20E.pdf)
